# Zearalenone Degradation by Dielectric Barrier Discharge Cold Plasma: The Kinetics and Mechanism

**DOI:** 10.3390/foods11101494

**Published:** 2022-05-20

**Authors:** Zhe Zheng, Yousheng Huang, Liping Liu, Yi Chen, Yuanxing Wang, Chang Li

**Affiliations:** 1 State Key Laboratory of Food Science and Technology, China-Canada Joint Laboratory of Food Science and Technology (Nanchang), Nanchang University, Nanchang 330047, China; zhengzhe1121@163.com (Z.Z.); chenyi-417@163.com (Y.C.); yuanxingwang@ncu.edu.cn (Y.W.); 2 Jiangxi Institute of Analysis and Testing, Nanchang 330029, China; yshuang0526@163.com; 3 College of Water Conservancy and Ecological Engineering, Nanchang Institute of Technology, Nanchang 330099, China; liuliping1997@126.com

**Keywords:** DBD cold plasma, zearalenone, degradation kinetics, degradation products

## Abstract

In this study, dielectric barrier discharge (DBD) cold plasma was used to degrade zearalenone and the efficiency of degradation were evaluated. In addition, the degradation kinetics and possible pathway of degradation were investigated. The results showed that zearalenone degradation percentage increased with increasing voltage and time. When it was treated at 50 KV for 120 s, the degradation percentage could reach 98.28%. Kinetics analysis showed that the degradation process followed a first-order reaction, which fitted the exponential function model best (R² = 0.987). Meanwhile, liquid chromatographywith quadrupole time-of-flight mass spectrometry (Q-TOF LC/MS) was used to analyze the degradation products, one major compound was identified. In this study, the reactive species generated in cold plasma was analyzed by Optical Emission Spectroscopy (OES) and the free radicals were detected by Electron Spin Resonance (ESR). This study could provide a theoretical basis for the degradation of zearalenone to a certain extent.

## 1. Introduction

**Figure 1:**
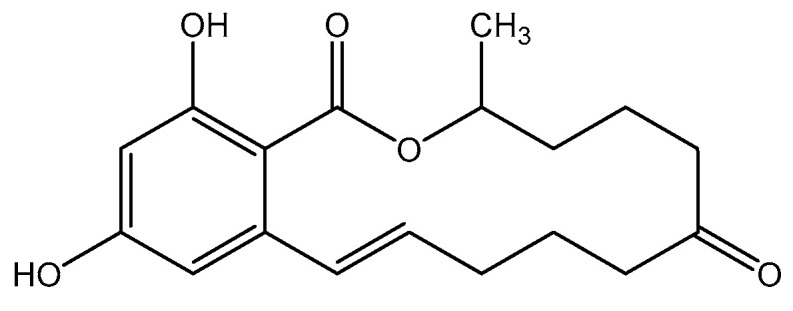
Chemical structure of zearalenone.

Mycotoxins are secondary metabolites of fungi wtih low molecular weight, potentially toxic to human and animal even at low concentration [1,2]. Zearalenone (Figure 1) is an estrogenic mycotoxin produced by Fusarium, which is one of the most widespread mycotoxins contaminating foods [3]. Zearalenone can be detected in corn, wheat and other grains and their cereal products [4]. Previous research has reported that the toxicity of zearalenone can be segmented into reproductive toxicity, genetic toxicity and immunotoxicity [5]. Zearalenone can participate directly in and interfere with the reproductive process, causing oxidative stress and heat stress, leading to cell and DNA damage, and even resulting in cell apoptosis [6]. Whether exposure to zearalenone via cereals or indirectly via feed and animal-derived products, all represent a significant threat to both human and animal health [7].

At present, the degradation methods of zearalenone mainly include physical method, chemical method and biological method [8]. Traditional physical and chemical methods may decrease the nutritional quality of food and palatability of feed, or leading to residues and safety concerns [9]. Therefore, efficient approaches for decontamination of zearalenone in food and feed are urgently needed. Biological method has the advantages of mild reaction conditions, no secondary pollution and relatively lower cost, but it is not suitable for food processing [10]. 

Cold plasma as a new non-thermal treatment technology received much attention in recent years. Cold plasma is a kind of partially or wholly ionized state of gas and composed of highly reactive species including gas molecules, charged particles in the form of positive ions, negative ions, free radicals, electrons [11]. Cold plasma has been used in various fields such as destroying enzyme activities, modifying food matrix features, degrading toxins and reducing pesticide residues, which can provide a new method for non-thermal processing and preservation of high-value agricultural products [12]. In recent years, more and more studies choose cold plasma as the degradation method of mycotoxin [13,14]. However, the application of cold plasma in zearalenone degradation research is relatively less and not deep enough [13,14]. Therefore, it is of great significance to investigate the degradation effect of zearalenone by cold plasma treatment. 

In the present study, DBD cold plasma was utilized for the degradation of zearalenone in different parameters. The content of zearalenone was determined by LC-MS/MS and the degradation kinetics was investigated at meantime. In addition, the degradation products of zearalenone were analyzed by Q-TOF LC/MS. OES was employed to detect the reactive species in plasma. ESR spectroscopy was applied to identify the free radicals in the plasma. This study provides a theoretical basis for the degradation of zearalenone by cold plasma. 

## 2. Materials and Methods

### 2.1. Materials

Pure zearalenone standards in acetonitrile (50 mg/L) were purchased from ANPEL (ANPEL Laboratory Technologies Co., Ltd., Shanghai, China). Zearalenone were diluted in acetonrtrile to obtain a concentration of 50 ng/mL and stored at −20 °C. 5,5-dimethyl-1-pyrroline-N-oxide (DMPO); 2,2,6,6-tetramethylpiperidine (TEMP, >98.0%) and N-tert-Butyl-α-phenylnitrone (PBN) were purchased from Sigma (Sigma Aldrich Co., Ltd., Shanghai, China). All of other reagents were provided by Aladdin (Aladdin Chemicals Co., Ltd., Shanghai, China).

### 2.2. Experimental Apparatus

**Figure 2:**
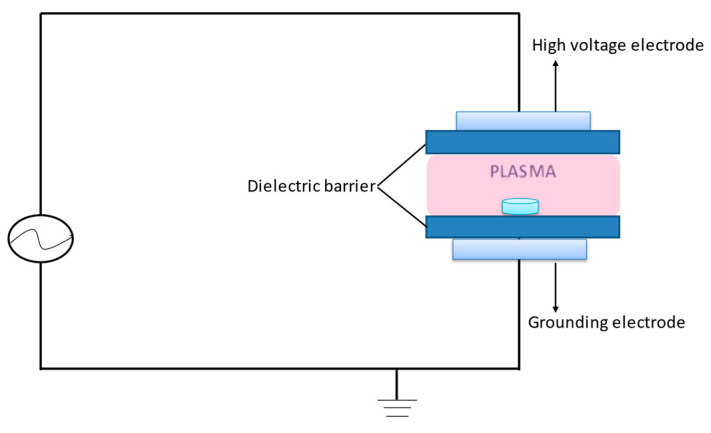
Schematic diagram of DBD plasma.

A schematic diagram of DBD plasma generator used in the present study is shown in Figure 2. The experimental apparatus essentially comprises a high-voltage alternating current power source, two copper electrodes and two dielectric boards. The work gas was atmospheric air. Small plastic jars were used as reactor. 

### 2.3. Treatment of Zearalenone with Different Plasma Parameters

1 mL zearalenone standard solution (50 ng/mL) were added into a plastic container and placed in the dark at room temperature. After 30 min, when solvent evaporated to dry, the sample was subjected to cold plasma treatment at different parameters. The samples were treated with three treatment voltages (30, 40 and 50 KV) for 12 treatment time (10, 20, 30, 40, 50, 60, 70, 80, 90, 100, 110 and 120 s), respectively. Throughout the experiments, two PVC boards with 2 mm thickness were used as the dielectric materials, and the distance between the two parallels boards was 2 cm. After the plasma treatment, samples were redissolved in 50% (*v*/*v*) acetonitrile water solution. Each treatment was performed on three sample replicates.

### 2.4. Degradation Efficiency Determination

Zearalenone were quantified with LC-MS/MS (Agilent Technologies, Santa Clara, CA, USA), according to the official method described in National Standard for Food Safety, Determination of Zearalenone in Food of the People’s Republic of China [15]). The standard solution of zearalenone (50 mg/L) was prepared in acetonitrile at concentrations at 1, 5, 10, 25, 50 ng/mL, respectively, and make a standard curve. Zearalenone concentration was calculated from the detector response to injected samples using a standard calibration curve. All chromatographic separations were performed using a C_18_ column (2.1 × 50 mm, 1.8-Micron); mobile phase was (A) acetonitrile (*v*/*v*), (B) 0.1% formic acid in water (*v*/*v*). Sample injection volume was 5 µL. Isocratic elution was performed with mobile phase A and B in the ratio 10:90, the time was 9 min. The flow rate was 0.3 mL/min. The UPLC system was coupled to triple quadrupole mass spectrometer with electrospray ion source (ESI) and the detection were performed in positive ionization mode. Degradation percentage of zearalenone was calculated based on the initial concentration of zearalenone using the following equation:

$$\mathrm{Degradation}\;\mathrm{percentage}=\;\frac{C_{0}-C_{t}}{C_{0}}\times 100\%$$

Actual degradation amount = *C*_0_ − *C_t_*

where *C*_0_ is the initial concentration of zearalenones and *C_t_* is the residual concentration of zearalenone after cold plasma treatment.

### 2.5. Degradation Kinetics of Zearalenone

Experimental data of zearalenone degradation was modeled using a kinetics degradation model, the effect of plasma treatment time on the kinetics of zearalenone degradation can be evaluated. The processing voltage is 40 KV and the dielectric material is PVC board with thickness of 2 mm. The degradation percentage of zearalenone (y) was taken as the vertical coordinate and the reaction time (x) as the horizontal coordinate. For modeling the degradation kinetics of zearalenone, the experimental data were fitted by logarithmic, quadratic, cubic and exponential functions (S) by using the curve estimation in SPSS. The function model with the best fitting effect was selected. In addition, the relevant parameters of the kinetic equation were calculated.

### 2.6. Structural Elucidation of Degradation Products

In order to assess possible pathways of the degradation, the possible degradation products were analyzed employing the UHD Accurate-Mass Q-TOF LC/MS (Agilent 1290-6538, Santa Clara, CA, USA). The standard solution of zearalenone (1 µg/L) was prepared in acetonitrile, the sample was treated with 50 KV for 120 s. The chromatographic condition was same as described in Section 2.4, ESI^−^ was used for collecting signals. The pump was operated at a flow rate of 0.3 mL/min with mobile phases A, 0.1% formic acid in water (*v*/*v*) and B,100% acetonitrile with injection volume of 3 μL. A linear gradient was started with 10% B, after which B was increased linearly to 100% in 13 min, and subsequently kept isocratic for 3 min. The proportion of B was then decreased to 10% in 2 min. The total run time was 18 min. The ion source parameters for ESI in negative mode over the range *m*/*z* 50–1200. Principal component analysis power and cluster analysis were also performed in Masshunter.

### 2.7. The Active Species Diagnosis by Optical Emission Spectroscopy

Optical emission spectroscopy (OES, AvaSpec-Mini4096CL, Avantes Corporation, Apeldoorn, The Netherlands) was used to characterize some of the active species in the cold plasma. Briefly, the spectra of excited gas plasma were measured using optical emission spectroscopy over the entire wavelength range of 200–1100 nm. The slit width of was 10 μm and the optical resolution was 0.88 nm. PVC was selected as the dielectric material, the plasma treatment voltage were 35, 40, 45 and 50 KV, respectively. All spectra were corrected by subtracting the noise from the background scans. 

### 2.8. Free Radical Identification by Electron Spin Resonance (ESR)

Electron spin resonance (ESR, EMXnano, Bruker Corporation, Karlsruhe, Germany) was applied to detect free radicals in the liquid system treated by the cold plasma. DMPO was dissolved in ultra-pure water (0.5 mol/L) to trap hydroxyl radicals (•OH). TEMP and PBN were dissolved in toluene (0.3% PBN) to trap singlet molecular oxygen (^1^O_2_). 1 mL spin-trapping solution were exposed to the plasma at 50 KV for 60 s. The ESR microwave power was setat 3.16 mW, the microwave frequency was 9.62 GHz, the scan number was 20, and the sweep time of 30 s was used. Other parameters of the ESR spectrometer were set as follows: the central field was 3425 G, the sweep width was 150 G and the attenuation was 15 dB.

### 2.9. Statistical Analysis of Data

Statistical analysis was carried out using SPSS 23.0, and a significant difference was verified by one-way ANOVA with Waller Duncan’s multiple range test (*p* < 0.05).

## 3. Results

### 3.1. Treatment of Zearalenone with Different Plasma Parameters

**Figure 3:**
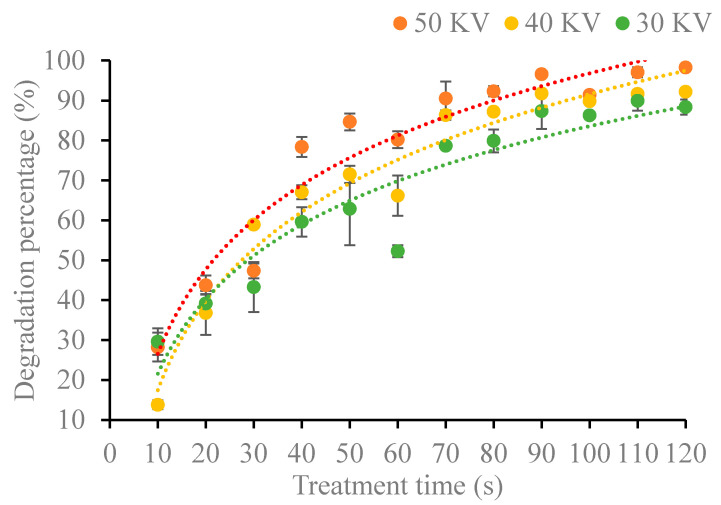
Degradation of zearalenone under different plasma treatment parameters.

Cold plasma can directly or indirectly decontaminate zearalenone. In this study, as shown in Figure 3, the degradation percentages of zearalenone increased with the treating time and tended to degrade completely by the end of the treatment. At the beginning of the study, there was no significant difference in degradation percentage at different voltage, which might be due to the short treatment time and zearalenone has not been sufficiently degraded. However, as the treatment time increased, such as the treatment with voltage 50 KV and the treatment time 60 s, the degradation percentage could reach 80%. Especially when the treatment time was 120 s, the degradation percentage could reach to 98.28%. When the treatment time was 40 s, zearalenone demonstrated a degradation of 59.62% at 30 KV, 67.06% at 40 KV and 78.37% at 50 KV, respectively. In the same way, when treated for 120 s, zearalenone demonstrated a degradation of 88.37% at 30 KV, 92.21% at 40 KV and 98.28% at 50 KV, respectively. These results confirmed that whether treatment time or voltage increased could lead to the increase of degradation of zearalenone.

According to Siciliano [16], with the increase in power and time, the residual AFB_1_ decreased from 25.4% to 0%. Devi [17] confirmed that the higher power could reduce the production of aflatoxins when Aspergillus parasiticus and A. flavus were treated with cold plasma. They considered that the phenomenon might be due to the high voltages could produce a higher number of radicals and charged particles, so it was better for mycotoxin degradation. Other studies have found that DBD cold plasma ionizes air through ultrahigh voltage, which is why voltage plays an important role in degradation efficiency [18]. The results showed that the reaction time of the active species in the cold plasma increased with the treatment time, the rate of reactive species generated increased with the increase of voltage. Thus, increasing the reactive species such as reactive oxygen species (ROS), free radicals and ultraviolet photons, when acting on the preservation of agricultural products, which allows more active particles to interact with mycotoxins and results in a higher degradation percentage [19].

### 3.2. Degradation Kinetics of Zearalenone

In previous studies, first-order model has always been used to model the inactivation kinetics of microorganism and enzyme by heating and other technologies [20].

**Figure 4:**
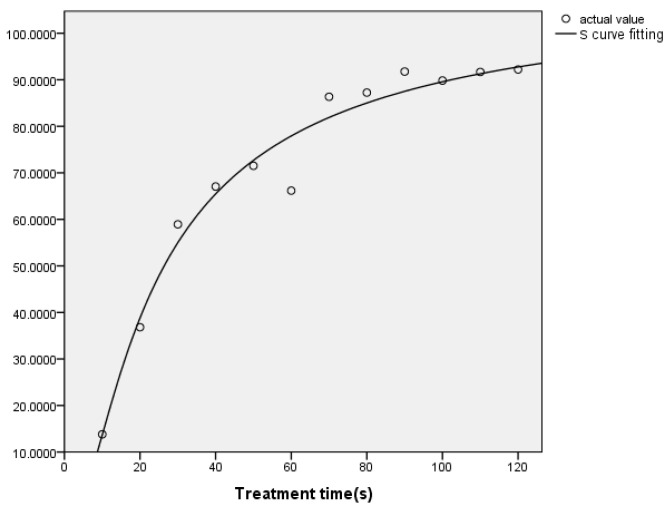
Effect of reaction time on degradation of zearalenone.

In the same way, the effect of plasma treatment time on the degradation of zearalenone was studied from the kinetic point of view. The degradation percentage of zearalenone varies with treatment time as shown in Figure 4. The first-order kinetics fitting was carried out using SPSS software, and four kinds of function models, namely logarithmic model, quadratic function, cubic function and exponential function, were fitted, respectively. The kinetics model parameters and correlation coefficients of zearalenone degradation are shown in Table 1.

The correlation coefficient of the four models (R^2^) were greater than 0.9, it can be observed that the first-order model was satisfactory with a high coefficient. In addition, ANOVA analysis showed that the regression model had statistical significance. The correlation coefficient of the exponential function R^2^ = 0.987 is close to 1, so it was chosen to express the degradation kinetics of zearalenone, the expression is $Y=e^{4.704-20.905/x}$, and the fitting effect is shown in Figure 4. The results showed that the degradation of zearalenone accorded with the first-order kinetics, which was similar to the previous results [21].

### 3.3. Structural Elucidation of Degradation Product

**Figure 5:**
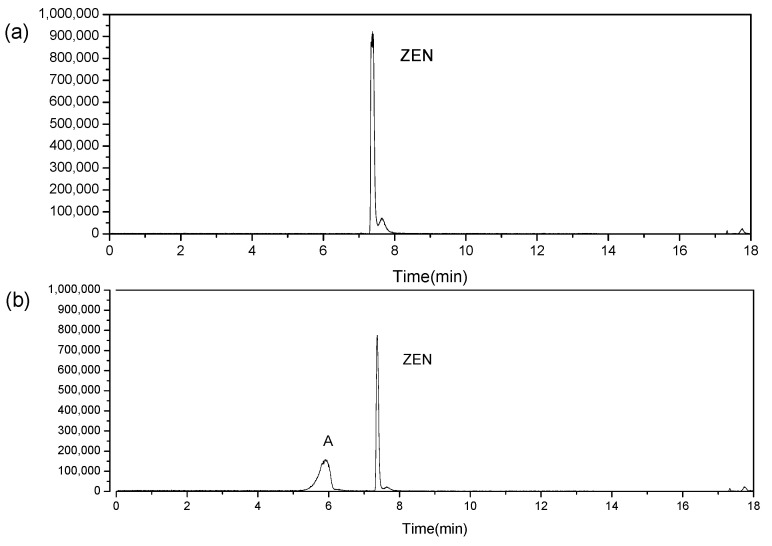
Chromatograms of zearalenone (ZEN) treated with cold plasma where (**a**) zearalenone control, (**b**) 1 µg/mL zearalenone after 120 s treated with detected degradation products.

**Figure 6:**
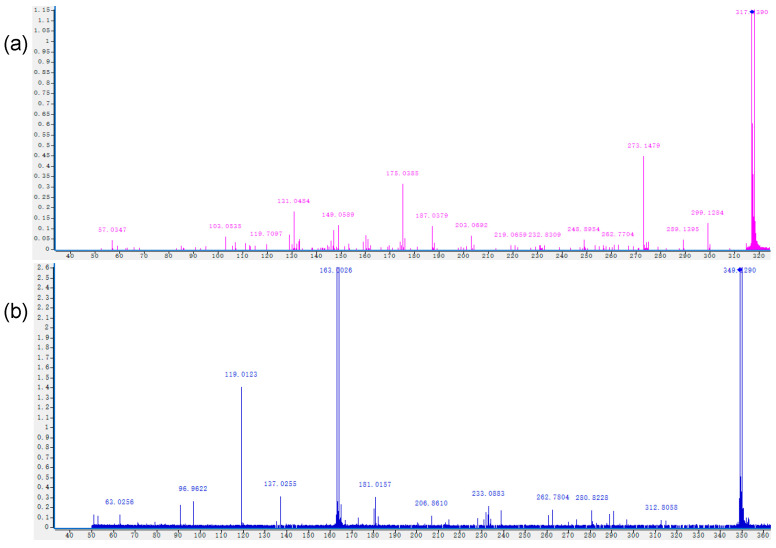
Mass chromatograms of zearalenone treated with cold plasma where (**a**) zearalenone control, (**b**) 1 µg/mL zearalenone after 120 s treated with detected degradation products.

The parent ions of zearalenone can be split α and β by mass spectrometry when they are bombarded by different energy levels, so the parent ions of the degradation products are deduced by ion splitting, and the possible structure of the ion fragments can be inferred. The analysis of the chromatograms confirmed a decrease of zearalenone, meanwhile one degradation product was found after cold plasma treatment (Figure 5). The molecular weight of zearalenone degradation products was calculated and analyzed with MassHunter Qualitative Analysis B.07.00 (Agilent Technologies, Santa Clara, CA, USA). In the secondary mass spectra as shown in Figure 6a, its reference peak is [M-H]^−^ at 317.1390 *m*/*z*. The main characteristic ion fragments are[M-H_2_O]^−^ −299.1284 *m*/*z*, [M-CH_2_O_2_]^−^ at 273.1479 *m*/*z*, [M-C_6_H_10_O_2_]^−^ at 203.0692 *m*/*z* and so on. And the secondary mass spectra of degradation product can be seen in Figure 6b. 

The molecular formula, mass-to-charge ratio and retention time of zearalenone and degradation product were list in Table 2. According to the above information, the molecular formula of degradation product was compared with zearalenone and the structure was speculated [22]. The degradation product observed at 349.1290 *m*/*z* as shown in Table 2 corresponded to a molecular formula of C_18_H_22_O_7_. 

**Figure 7:**
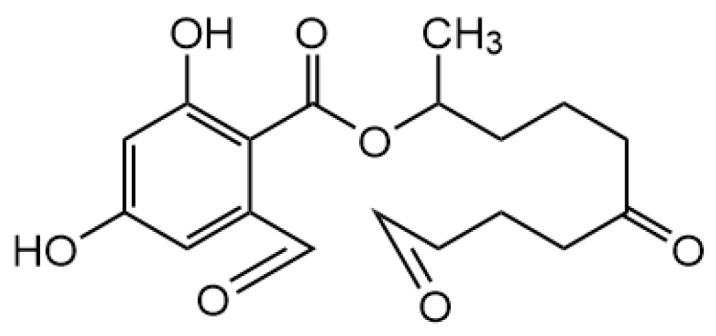
Chemical structure formula of zearalenone degradation product A.

**Figure 8:**
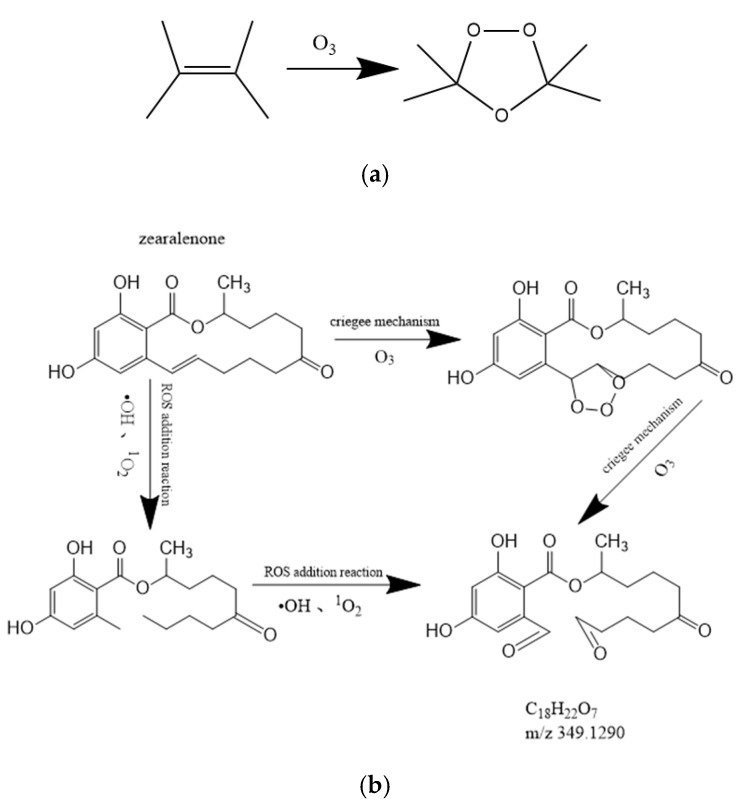
Degradation process where (**a**) criegee mechanism, (**b**) proposed pathway of zearalenone degradation.

Ozone was generated in cold plasma when the working gas was air [8]. Since the structure of zearalenone is cyclic olefin, it will continue to decompose into ring-opening compounds after cold plasma treatment, and the ozone generated by the system is added to the olefin double bond, which generates aldehydes at the ends of the double bond, resulting in product A (Figure 7). As shown in Figure 8a the zearalenone molecule could occurr ozonolysis, where ozone first underwent 1,3-dipole cycloaddition of the olefin to obtain the primary ozonide, rearranged to obtain the zwitterion peroxide (criegee zwitterion), and then another 1,3-dipole cycloaddition to produce the secondary ozonide. In this study, the C=C in the zearalenone molecule was broken by the oxidation of ozone to form 1,2,3-trioxane cyclic compounds. The possible degradation pathways are shown in Figure 8b. In addition, the broken of C=C may also be due to the collision of zearalenone molecules with oxidizing reactive species, such as ·OH and ^1^O_2_ under the effect of high field strength of cold plasma, which led to the broken of the C=C, followed by a radical addition reaction in which the reactive oxygen species in the system binds to the olefin double bond and generates aldehydes at the ends of the double bond, resulting in product A. The high electric field intensity in the plasma may lead to the decomposition of water molecules and the formation of ·OH. Especially ·OH as an oxidant, it is easy to die in the addition reaction with unsaturated bond [22]. In addition, some free electrons may be trapped in the solution and may occur dehydroxylation reaction [23].

### 3.4. The Active Species Diagnosis by Optical Emission Spectroscopy

**Figure 9:**
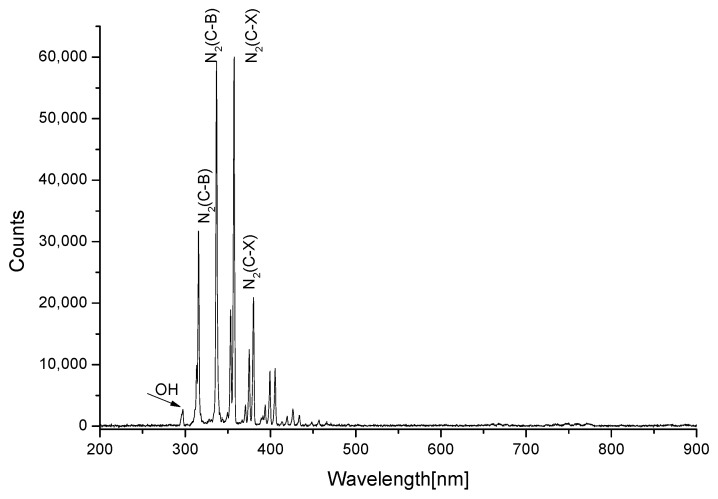
Optical Emission spectra of air plasma.

OES is generally employed for obtaining qualitative information about the type of reactive species in the plasma; for example, OES of air plasma often reveals the presence of excited nitrogen species, atomic oxygen, and hydroxyl radicals [24]. The emission spectra of air plasma is shown in Figure 9. The emission peaks were previously reported to be mainly attributed to N_2_, O, and OH species, when air was used as working gas [25]. It can be clearly observed that the emission spectrum is dominated by N_2_ second positive system (N_2_ (C-B)) at 300–430 nm. Since N_2_ is the major component of the air, and N_2_ (C-B) can be excited by direct or step-wise electron impact. O has not been detected in our study, but has been confirmed in other studies [26]. In addition, another study confirmed low intensity emissions from singlet O were noted at 758 nm and 844 nm [27]. The small peak of ·OH were recorded near 295–300 nm in this study, it was generated from moisture in air. These results indicated that the cold plasma in this study was an abundant source of ROS and RNS. 

From the emission spectrum, it is evident that the emission is in the near UV region (300–400 nm), which is similar to reported studies for DBD operating at atmospheric pressures in air [28]. This is also consistent with the results of previous studies monitored the plasma microjet operated in air. An optical emission spectrum was found in the range of 200–400 nm and very weak emissions was observed in the 200–300 nm range, which was likely attributed to molecular NO [29]. During cold plasma formation, the reactive species (ROS, RNS) of different wavelengths can cause the dissociation of the covalent bonds of zearalenone, thereby the original molecular structure was destroyed [30]. 

**Figure 10:**
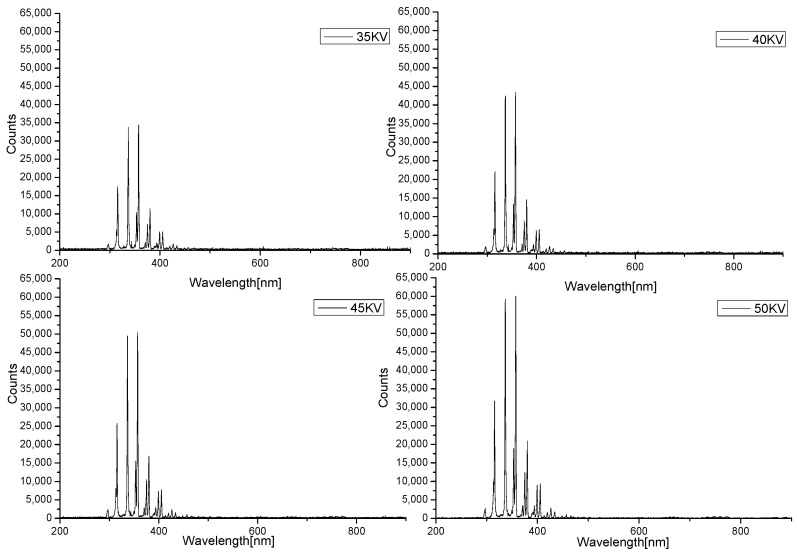
Optical Emission spectra of air plasma in different environments.

In addition, when PVC was used as the dielectric material, and air was used as working gas, the main reactive species information under 35 KV, 40 KV, 45 KV and 50 KV, respectively, were obtained by optical emission spectroscopy. As shown in Figure 10, the types of active particles obtained under different voltages are same, and with the improving of voltage, the intensity of the spectral signal increases. When the voltage was 50 KV, the signal strength reached more than 60,000 a.u. This result confirmed what we supposed in the previous section. As the operating voltage increased, more active particles were produced, the higher was the degradation rate of zearalenone.

### 3.5. Free Radical Identification by ESR

ESR is the most direct and effective technique for qualitative analysis of free radicals. Free radicals are very unstable and are easily to be quenched, so it is difficult to be detected directly in the reaction process by ESR. It is necessary to add a trapping agent to form a stable spin adduct, then the type of free radical can be qualitatively analyzed according to the spectrogram.

**Figure 11:**
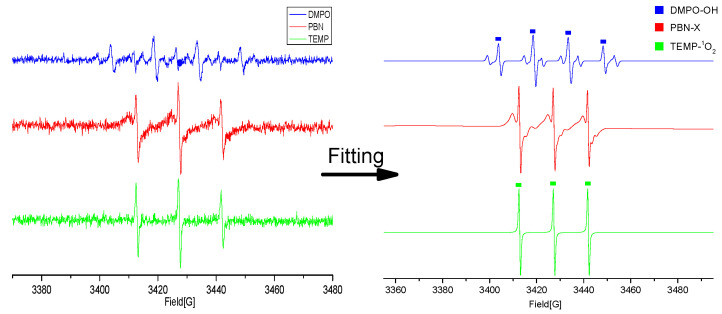
ESR spectra obtained by different trapping agents.

The spectra obtained from the experiment were fitted by the Xenon software (Figure 11). The direct spin trapping reaction between DMPO and ·OH produces spin adduct DMPO-OH, which is characterized by a quartet ESR spectrum with a peak intensity ratio of 1:2:2:1 as shown in Figure 9. DMPO-OH may also derive from the decay of DMPO-OOH, or the oxidation of DMPO by ^1^O_2_. Other small peaks are attributed to NO. In addition to the plasma treatment process that could produce NO, they were possibly produced by oxidation of DMPO itself in the reaction as well. Similarly, non-radical ^1^O_2_ and nitroxide radicals were trapped in some cases, usually ^1^O_2_ was trapped by TEMP [31]. The peak of spin adduct TEMP−^1^O_2_ was characterized by a quartet ESR spectrum with a peak intensity ratio of 1:1:1:1 [32]. As shown in Figure 11, the spectra of TEMP−^1^O_2_ were confirmed by the characteristic peaks of the spin adduct. The type of free radical captured by PBN is not specific, a single free radical type cannot be determined from the fitted spectrum of PBN adducts. In this study, the major radicals are probably H, N_3_, •OH. Previous study showed that the free radical and high energy electron produced by cold plasma could react with mycotoxin, which leaded to the breaking of chemical bond and the formation of low molecular weight degradation products [33]. In addition, Hu [34] proved that •OH radical played an important role in the DBD plasma degradation of dimethoate. •OH radicals attacked the P=S bond of dimethoate, forming the P=O bond; the intermediates further reacted with •OH radicals to produce the degradation products.

## 4. Conclusions

In this study, cold plasma as a non-thermal treatment technology was utilized to degrade zearalenone. The degradation percentage of zearalenone increased with the increase of treatment time and treatment voltage. The degradation of zearalenone accords with the first-order kinetics. The molecular formula of one degradation product was identified and verified. Generation of reactive species were characterized with OES and ESR. The data indicated that reactive species were also influenced by different working conditions. More reactive species were produced in cold plasma with higher discharging voltage. The degradation of zearalenone is attributed to reactive species such as ozone, free radicals and ROS formed in cold plasma.

At present, the toxicity of degradation products of zearalenone is still not clear. Therefore, future studies should be focused on the toxicity of degradation products further by cell or animal model experiments.

## Figures and Tables

**Table 1:** Parameter and R² value of reaction kinetic model of zearalenone degradation.

Model	Equation	F	R^2^	Sig.
Log function	Y = 32.204 logx − 56.677	242.414	0.956	<0.001
Quadratic function	Y = 4.904 + 1.737x − 0.009x^2^	80.380	0.935	<0.001
Cubic function	Y = 2.715x − 0.027 x^2^ + 9.274e^−5^x^3^ − 7.755	66.893	0.947	<0.001
Exponential function (S)	$Y=e^{4.704-20.905/x}$	812.195	0.987	<0.001

**Table 2:** Mass accuracy measurement using LC–Q-TOF–MS for zearalenone and its degradation product.

Compound	Formula	Observed *m*/*z*	Retention Time (min)	Mass Error (ppm)
Zearalenone	C_18_H_22_O_5_	317.1390	7.450	1.41
A	C_18_H_22_O_7_	349.1290	5.839	0.79

## Data Availability

Not applicable.
